# Digital transformation of robotic surgery train the trainer ‘TTT’ courses: training the trainer in technique and technology (the 4Ts course)

**DOI:** 10.1007/s11701-025-02685-8

**Published:** 2025-08-25

**Authors:** Justin W. Collins, Nader Francis, Fares Haddad, Mark Coleman, Steven Stamenkovic, Asit Arora, Bas Wijnhoven, Altaf Awan, Tom Cecil, Nahid Gul, Jawad Ahmad, Subash Vasudevan, Ben Challacombe, Nuha A. Yassin

**Affiliations:** 1 Division of Surgery & Interventional Science, University College London, London, UK; 2 Division of Uro-Oncology, University College London Hospital, London, UK; 3 University College London, London, UK; 4 University Hospitals Birmingham NHS Foundation Trust, Birmingham, UK; 5 The University of Birmingham, Birmingham, UK; 6 The Royal College of Surgeons of England, London, UK; 7 The Griffin Institute, Northwick Park, Harrow, UK; 8 St Mark’s Hospital, London, UK; 9 Department of Surgery, University Hospitals Plymouth NHS Trust, Plymouth, United Kingdom; 10 Thoracic surgery, Barts Health NHS Trust, London, United Kingdom; 11 ENT surgery, Guy’s and St Thomas’ NHS Foundation Trust, London, United Kingdom; 12 Oncological and Gastrointestinal Surgery, Erasmus University Medical Center, Rotterdam, Netherlands; 13 Division of General Surgery, Derby Hospitals NHS Foundation Trust, Derby, United Kingdom; 14 Colorectal surgery, Hampshire Hospitals NHS Foundation Trust, Basingstoke, United Kingdom; 15 Obstetrics and gynaecology, Wirral University Teaching Hospital NHS Foundation Trust, Wirral, United Kingdom; 16 Hepatobilary surgeon, University Hospitals Coventry & Warwickshire NHS Trust, Coventry, United Kingdom; 17 General surgery, East Suffolk and North Essex NHS Foundation Trust, Colchester, United Kingdom; 18 Department of urology, Guy’s and St Thomas’ NHS Foundation Trust, London, United Kingdom

**Keywords:** Robot-assisted surgery, Training, Curriculum development, Train the trainer, Hydrogel models, Virtual Reality, Surgical education, Digital Transformation, Surgical Data Science

## Abstract

As the role of robot-assisted surgery continues to expand there has been as an associated proliferation of novel technologies to aid training. Necessitating the development of standardised and validated training programmes that incorporate guidance on curriculum development with training techniques, and where and when to utilise novel training technologies. To provide guidance on an optimised “Train-the-Trainer in Technique and Technology” (4Ts) structured educational programme for surgical trainers, in which delegates learn a standardised approach to training candidates in knowledge and skills acquisition both for surgery and the utilisation of novel training technologies. We aim to describe a 4Ts course for robotic surgery based on the current published literature and to define the key elements within a 4Ts course by seeking consensus from an expert committee formed of key opinion leaders in training and MedTech industry. The project was carried out in phases: a systematic review of the current evidence was conducted, a hybrid meeting was held, and an initial survey was created based on the current literature and expert opinion and sent to the committee. Twenty experts in robotic training, contributed to the Delphi process that included clinicians, academics, and industry representing nine different surgical specialties and seven different robotic companies. An accelerated Delphi process underwent three rounds of survey in total. Additions to the second- and third-round surveys were formulated based on the answers and comments from the previous rounds. Consensus opinion was defined as 80% agreement. There was 100% consensus that there was a need for a standardized platform agnostic 4Ts course in robotic surgery. A consensus was reached in multiple areas, including the following: (1) definitions and terminologies, (2) qualifications to attend, (3) course objectives, (4) pre-course considerations, (5) requirements of e-learning, (6) theory and course content, and (7) measurement of outcomes and (8) performance certification and regulation. The resulting formulated curriculum showed good internal consistency among experts, with a Cronbach alpha of 0.90. An evidence-based consensus has been achieved to reach content validation for guidance on a 4Ts curriculum for robotic surgery training. This recommended content lays the foundation for developing platform agnostic metric-based progression curricula for trainers in robotic surgery. Future 4Ts curricula related to procedural training will require further validation. As the role of robot-assisted surgery continues to expand, development of standardised and validated training programmes is becoming increasingly important. There is currently a lack of agreement on how best to train trainers in both training and awareness of novel training technologies. We report a consensus view on a standardised “4Ts” curriculum focused on robotic surgery. It was formulated by polling the opinions of experts and industry, combining current evidence for training technologies with experts’ knowledge of surgical training.

## Introduction

Adoption of robotic systems for minimally invasive surgery has rapidly increased during the last 20 yrs [1]. With the more recent emergence of multiple robotic platforms there is a developing need for platform agnostic training and better options to compare platforms in different surgical specialties and to share expertise across platforms [2].

Surgical training continues to evolve from the traditional Halstead approach of ‘see one, do one, teach one’ [3]. With the introduction of more complex MedTech surgical tools, that required instructions for use, and a better understanding of the benefits from structured and standardised curricula development that enabled deliberate practice [4], new approaches to surgical training were sought that aimed to avoid naïve practice. Sharing experts’ insights, knowledge and skills on successful implementation of robotic surgery would shorten learning curves and avoid discovery curves and their associated patient harm [5]. This transformation was part realised with the emergence of wet-lab training hubs, that were able to centralise expertise. But training is expensive to deliver with additional costs related to the need for industry to donate hardware that cannot be utilised for direct patient care, and further additive costs of travel, accommodation and time away from the workplace. Additionally, simulation models on animals lack relatable anatomy and human cadavers lack pathology to address crucial surgical skills learning objectives.

There are other elements to training that need to be urgently considered. There are huge disparities in access to surgical expertise and training. The Lancet-commissioned review on Global Surgery 2030 concluded that nearly five billion people do not have access to surgical care. It is estimated that the cost to train the identified shortfall of 2.2 million surgeons and anaesthetists required for low middle income countries (LMIC) to have parity, is $350 billion [6]. However, disparities are not limited to LMIC and quality performance reporting following surgery has highlighted disparities in the quality-of-care patients receive in the USA [7]. As awareness of the importance and benefits of training increase, current training methodologies such as wet-lab centres have become a cost–benefit bottleneck to access training.

To address the need for better training delivered by trainers, guidance was published in 2019 on a metrics-based approach to a ‘Train the Trainer’ (TTT) course, that was formulated and validated using the Delphi process [4]. However, this publication did not address the need for equity in training access [8]. In recent years there has been an increased focus on the importance of both benefits of training and improved access to standardised validated approaches, and this has resulted in the third wave of surgical training’s evolution with digital transformation. Novel training technologies include Virtual Reality (VR) simulation, hydrogel models and telepresence services, whilst digital training technologies incorporate automated objective performance metrics, personalised feedback, data sharing and predictive AI analytics [9, 10]. Digitalisation enables wider access to standardised training curricula that incorporate agreed benchmarked objective metrics of performance, resulting in options to access expertise in more scalable and cost-efficient ways. Telesurgical communication further enables access to expertise with shared insights, knowledge and skills on a global scale [11]. However, rapid introduction of novel technologies necessitates training our trainers in both technique and the technologies available.

A digitalised train-the-trainer in technique and technology (4Ts) course, incorporating education on when and where to utilise novel training technologies, has potential to address 3 crucial aspects of optimised metrics-based training: namely global access to expertise, whilst maintaining standardisation and quality assurance through agreed benchmark assessments. With the Royal College of Surgeons of England, we concluded that a robotic platform agnostic 4Ts consensus meeting would be of benefit to define both the key pedagogy training elements and identify important novel technologies to be integrated. Our aim was to seek consensus from a specialist panel formed of professionals with expertise in training from healthcare and MedTech industries to give guidance on future curricula content development that includes objective metric-based assessments to improve equity in training outcomes.

## Evidence acquisition

The project was carried out in three phases:

1. A steering group was formed to review the literature and summarise the current evidence for TTT programmes in robotic-assisted surgery.
2. A larger expert panel convened and discussed the important aspects of a TTT programme based on the current evidence. Following presentations and open discussion, a survey was created, with the input from the panel members.
3. Thirdly, panel-based consensus findings were determined using an online Delphi process to formulate guidance and provide recommendations for future research.

## Review of the literature

A systematic review was performed in accordance with the Preferred Reporting Items for Systematic Reviews and Meta-analyses (PRISMA) statement [12]. In February 2025, we undertook a comprehensive computerised search using PubMed and Medline databases. We systematically searched using medical subject headings, including “robotic-assisted surgery training”, “robotic surgery training”, “curriculum development”, “train the trainer”, and “proficiency-based training”. The literature review was updated in May 2025.

Articles of interest included prospective studies on the impact of robotic training, robotic training curriculum development with validation, and systematic reviews on robotic training published between July 2000—when the first robotic systems received FDA approval in the USA [13] and May 2025. Other significant studies cited in the reference list of selected papers were evaluated, as well as studies of interest published after the systematic search to allow for a comprehensive review.

**Fig. 1:**
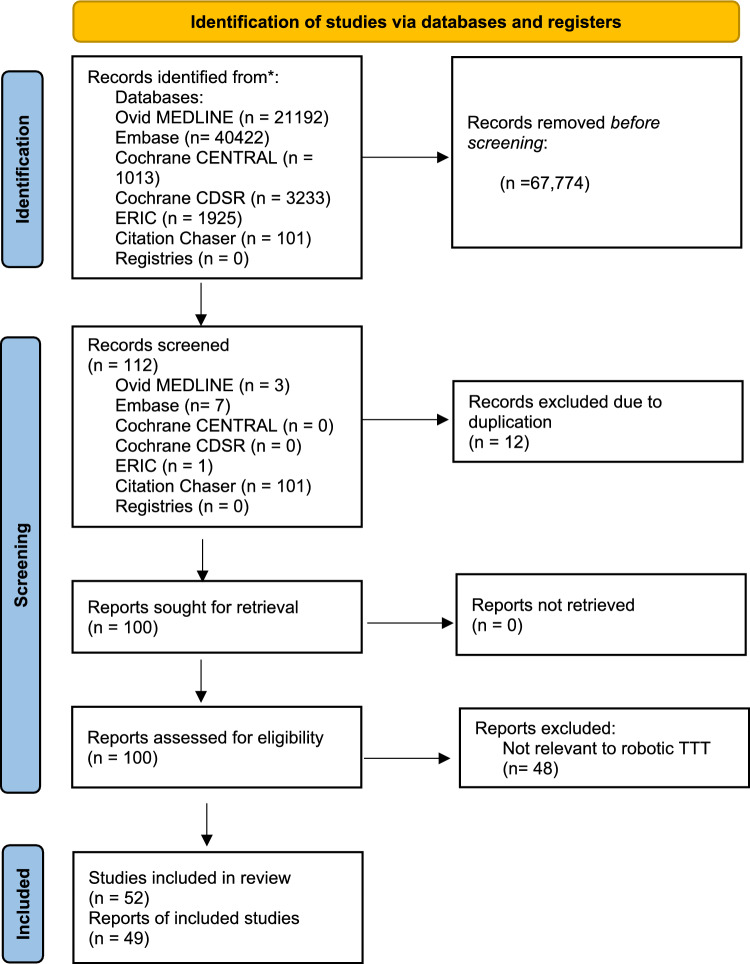
Flowchart of the selection of papers for the literature review on robotic training

Two reviewers independently selected papers for detailed review (N.Y. and J.C.) evaluating the abstracts and, if necessary, the full-text manuscripts. Potential discrepancies were resolved by open discussion. The electronic search yielded a total of 52 potential articles. Figure 1 summarises the PRISMA selection process. Overall, the quality of available studies was moderate to low [14]. Most of the available evidence consisted largely of expert opinion, consensus statements, and small qualitative studies.

### Evidence synthesis

The systematic review of 52 studies reveals four dominant Train-the-Trainer models that have emerged in robotic surgical education, each addressing distinct aspects of instructor development while facing unique regional adaptation challenges. The findings of the review are summarised in Table 1.

**Table 1:** Comparison of Train-the-Trainer (TTT) Models in Robotic Surgery

Model	Key features	Validation study	Advantages	Regional challenges
Preceptorship Cascade	Tiered certification (Master → Regional trainers); 6-month mentoring phase	Mottrie et al. [15]	High standardization (92% skill retention at 1 yr)	Requires sustained funding for preceptor travel
Simulation-Based	Trainers must achieve top 10% VR scores; monthly fidelity assessments	Boal et al. [16]	Reduces skill variability by 41%	Limited applicability in low-resource settings
Telementoring	Cloud-based platforms with 5G-enabled real-time feedback; quarterly audits	Nathan et al. [17]	Cuts costs by 60% vs. in-person	Latency issues in 34% of rural sites
Dual-Certification	Combines technical + pedagogical testing; uses standardized teaching scenarios	Tou et al. [18]	78% inter-rater reliability	Time-intensive (avg. 8 months per trainer)

### Expert panel

An advisory panel was formed, which comprised societal leads, global key opinion and industry leaders with a specialist interest in robotic surgery training and communication in education and training. In total, 20 experts from Europe and the US were brought together to discuss and develop guidance on a digitalised train the trainer curriculum with integration of novel training technologies, related to the 8 areas of interest. For the clinical academics contributing the medians (range) for panel members’ h index and i10 index were 30 (5–91) and 58 (5–422), respectively. The panel hybrid meeting was chaired by J.C and N.Y. with presentations on the current evidence.

### Internet survey and Delphi process

Following the hybrid meeting, the Delphi process was conducted to drive consensus among the experts. An Internet survey (Google forms) was generated based on the current literature and expert opinion and sent to the 20 committee members. The Delphi was divided into eight sections. The Supplementary material shows a full list of the survey questions. An accelerated e-consensus reaching exercise, over 3 consecutive days, using the Delphi methodology was then applied. The Delphi method structures group communications so that the process is effective in allowing a group of individuals to deal with a complex problem. Questions in which there was 80% consensus were removed from the next round of the survey. Repeated iterations of anonymous voting continued over three rounds, where an individual’s vote in the next round was informed by knowledge of the entire group’s results in the previous round. To be included in the final recommendations, each survey item had to have reached group consensus (80% agreement) by the end of the three survey rounds. In the Delphi process, the finding of “consensus” is more relevant than the level of consensus. Reliability of the formulated guidance was evaluated using Cronbach alpha to assess internal consistency among experts. Levels of consensus are reported in the Supplementary material.

## Results

### Formulation of guidance

We had 100% (20/20) response rate in all three rounds of the Delphi. There was high inter-rater reliability, which was > 0.80. After three rounds of Delphi surveys, a consensus was obtained in more than 80 elements in seven different categories. The categories were the following:

Section 1: A consensus on terminology.

Section 2: Prerequisites for 4Ts course selection and trainer qualifications.

Section 3: Objectives and focus of a 4Ts course.

Section 4: Pre-course considerations.

Section 5: Theory and course content.

Section 6: Measuring outcomes.

Section 7: Education and Integration of novel technologies to aid surgical training.

Section 8: Regulation and credentialing.

There was 100% agreement within the panel that there is a clear need for a 4 T’s course in robotic surgery that is platform comprehensive/agnostic with respect to foundational knowledge and the need to raise awareness of novel technologies in robotic surgery. There was also 100% agreement that to address current shortfalls in access to surgical training there is a need for guidance on a TTT curriculum online, that follows the FAIR principles of being Findable, Accessible, Interoperability and Re-usable [8].

#### *Section 1: Terminology*

Uniform communication language is important for understanding roles in surgical training. If there is ambiguity in the “surgical training” terminology, it may have implications in various clinical settings, for example, understanding who has responsibility regarding clinical decision making, trainer-trainee working relationships, communication between team members, with all ultimately impacting patient care. At the meeting, we presented and discussed important terminologies related to a 4Ts course. A summary of these terms based on previous guidance was circulated [4] and modifications and additions related to a 4Ts course agreed by the committee and can be seen in Table 2.

**Table 2:** Clarifications on terminology

Term	Definition
Outcome measures	Quantifiable consequences of an action, set of actions, or procedure; also the final result based that is to be measured (time, speed, accuracy, performance errors, leadership, communication skill)
Metric	Operational definition of an entity, object, location, etc.; must support an outcome measure (“no measurement, no metric”)
Benchmark	A defined point of reference against which things may be compared; it is to be noted that this is not the score
Training	Act of teaching a particular skill or type of behaviour (the faculty member actively instructs)
Assessment	Evaluation of the nature, ability, or quality of performance (the faculty only observes; if feedback given, then it is considered training)
Education	Culture or development of personal knowledge—understanding, growth of character, moral and social qualities, etc
Knowledge	The fact or state of having a correct idea or understanding of something; possession of information about something
Skill (specifically psychomotor skills)	Capability of accomplishing something with precision and certainty; ability to apply practical knowledge in defined and agreed way; ability to perform a function acquired or learned with practice
Mentor	A person who acts as a guide and an adviser to another person, especially one who is less experienced; a person who offers support and guidance to another; an experienced and trusted counsellor or friend
Trainer	A person who provides sustained instruction to perform particular skills, tasks, or functions
Preceptor	A person who gives instruction (in medical field, a physician or other health professional who gives practical clinical training to a medical student, nurse, etc.)
Proctor	A person responsible for supervising students in written examinations (originally at a university); an invigilator. A proctor in the setting of surgical training indicates someone who is not able to take over the operating in the OR
Simulation	An artificially created or configured “learning” situation that allows for the practice or rehearsal of all or salient aspects of a procedure, including the opportunity to enact both appropriate and inappropriate learner actions (i.e., errors); simulation should afford reliable and valid metric-based assessment of performance; assessments must, at a minimum, allow summative but preferably formative feedback on procedure performance proximate to task execution, particularly for metric errors [19]
Training	Sustained instruction and practice (given or received) in an art, profession, occupation, or procedure, with a view to proficiency in it; also physical preparation given or received
Practice	To exercise oneself in a skill or art to acquire or maintain proficiency, especially in music
Deliberate practice	Deliberate practice is a method of learning and skill development that focuses on purposeful, structured practice with the specific goal of improving performance. It involves conscious effort, focused attention, and feedback to refine skills in a systematic way
Curriculum	Content and specifications of a course of study
Device training	Education and instruction provided to users, on how to properly and safely operate and maintain medical devices. This training ensures proficiency in using the device for its intended purpose, minimizing risks of misuse or malfunction. Includes optimisation of setup and alarm management
Basic skills training	Structured, metric-based curriculum designed to train surgeons in the basic technical skills required for robotic-assisted surgery. It aims to equip surgeons with the knowledge and skills to safely and effectively perform defined tasks, to provide the foundational skills required to commence procedural training
Procedural training	The learning process that equips surgeons with the specific skills and knowledge needed to perform a particular robotic surgery procedure. It builds upon foundational robotic skills and progresses through observation, case assisting, supervised console training, and independent practice
Competent	A person having the necessary ability, knowledge, and skill to do something successfully
Proficient	A person with a high degree of skill or expertise; an operational definition of proficient is “that it is what proficient individuals do” [20]
Expert	A person who is very knowledgeable or skilful in a particular area
Test	A procedure intended to establish the quality, performance, or reliability of something, especially before it is taken into widespread use
Benchmarked assessment	A test that has defined objective criteria for pass or fail (often conducted by an independent, external body)
Certification	Confirmation that a certain level of achievement has been reached (awarded by an accredited authority after agreed and defined benchmarked assessment test)
Refocused/ Remedial training	Additional instruction provided for trainees who have been identified as requiring additional training or practice, because of poor performance
Surgical data science	The scientific discipline that seeks to improve the quality, safety, and value of interventional healthcare through the acquisition, organization, analysis, and modelling of data

The panel also identified that certain important descriptive terms currently have no clear consensus agreement. This TTT curriculum terminology was included in the Delphi process. The panel agreed that the surgeon being educated for the future role as a trainer is called a “trainer delegate” in the TTT course and that the person giving training on a TTT course is called the “lead trainer”. Only after successful completion of the 4Ts course, is the delegate is verified as a “trainer”.

#### *Section 2: Prerequisites for 4Ts course selection and 4Ts qualifications*

The panel reached a consensus view that TTT delegates should be experts in their fields and that there should be defined selection criteria for being accepted into a TTT course. There was 100% agreement that delegate trainers should be objectively assessed and certified with a metric-based assessment, to defined benchmark, on both the subject matter of the topic they are teaching, as well as the 4 T’s course. The panel concluded that important individual qualities for surgical trainers include being knowledgeable, interested, and enthusiastic; enjoying training; having sufficient case volume; being a good communicator; being supportive; having time to train; and knowing when to safely interject to prevent harm without trying to take over. Trainers need to be able to commit to a defined number of training courses per year. Recommendation for delegates for the 4Ts course may come from various sources: professional societies or organisations and hospital/programme directors Industry recommendations for proposing delegates did not reach consensus. When targeting delegates for the 4Ts course, selection should be linked to faculty development. The panel agreed that not all expert surgeons are automatically good trainers. The operating room is a unique educational setting that can require unique training skills [21]. Required attributes, knowledge and skills to optimise training, may also vary within different training settings. It has previously been shown that trainees and trainers have different perspectives on the attributes that make a good surgical trainer [22]. The literature review identified descriptions of the attributes of expert teachers. Hatem et al. [23] published a comprehensive list of the educational attributes of effective medical educators which are summarised in Table 2. Elements considered to further benefit from data driven and digitalised training are highlighted in green (Table 3).

**Table 3:** Attributes, knowledge, and skills of competent teachers and the impact of digitalisation (table based on Hatem et al. [23])

Attributes	Knowledge	Skills
Believes in a teacher's code of ethics for teaching medicine	Demonstrates awareness of and tacitly or explicitly employs basic pedagogic principles	Questions, listens, and responds effectively
Demonstrates passion as a teacher	Displays awareness of and uses teaching techniques in line with current neuroscience and cognitive psychological findings	Is a reflective, mindful teacher
Demonstrates kindness in all interactions	Is knowledgeable and up to date in one's discipline	Is able to capture and maintain attention
Manifests and stimulates curiosity	Promotes scholarship	Communicates knowledge effectively and makes it relevant to the learner
Values and functions as an effective role model	Understands the concept of creating a safe environment for the trainee	Demonstrates leadership in educational settings
Acknowledges that the goal of effective teaching is directed at effective learning and understanding		Demonstrates the basic skills for effective lecturing and facilitating small and large group discussions
Advocates for education and access to expert knowledge and skills		Establishes a learning community that values education and the process of continual learning
Demonstrates awareness of own limitations and is not afraid to say, “I don’t know”		Establishes an educational contract with learners, identifying learners’ needs and clarifying the teacher's expectations
Is accessible to learners		Gives praise as well as critical feedback in a manner acceptable to the learner
Seeks and obtains knowledge of learners		Is adaptable and flexible
Values and establishes a safe learning environment		Promotes critical thinking
		Promotes self-directed learning
		Provides timely summative evaluations
		Uses information technology effectively

#### *Section 3: Objectives of a TTT course*

The panel agreed that there should be clearly defined objectives for a 4Ts course and that the course should provide education addressing cognitive, technical and non-technical skills training [4]. The focus for a 4Ts course should incorporate a combination of setting up a training program, educating the trainer on training skills as well as raising awareness about novel training technologies that are relevant to their training and how to integrate and utilise data from these training technologies.

Key topics identified by the panel included key skills of an effective trainer and the principles of learner-centred training. The importance of setting clear learning goals, optimising training environments, providing guidance on defined technical skills and optimising feedback and debriefing. Formative feedback, performance enhancing feedback and the SMART feedback structure (Specific; Measurable; Achievable; Relevant: Timeframe) were highlighted as important [24].

The panel also recognised the need for rating and calibration exercises on training in both laboratory/simulation and in the OR and education on the importance of psychometric robustness of the various available training skills assessment tools. Other important areas of knowledge included highlighting the importance of the team’s contribution to training, defining the learning curve in surgery and understanding how to “shorten” the learning curve [25]. There was also agreement on the importance of being aware of the impact of stress in the training environment and the necessity for prioritising patient safety, during training [26]. Expectations and training goals have been shown to differ between the trainer and trainee [22]. To avoid misunderstandings, the panel recommended that the trainer should always seek agreement with the trainee regarding the goals and objectives of the course (trainee-directed learning).

The key to demonstrating values and benefits of a 4Ts course is showing evidence of successful faculty development that impacts training [27]. The panel concluded that important outcome measures from a 4Ts programme implementation indicating success included: shortened learning curves, increased theatre efficiencies, improved patient safety and avoidance of errors.

#### *Section 4: Pre-course considerations*

The panel considered what needs to be included in a “checklist” of basic requirements for setting up a 4Ts course. Multiple elements reached ≥ 80% consensus and are summarised in Table 4.

**Table 4:** Checklist for setting up a 4Ts course

Personnel	Facilities and equipment	Course content materials
A ‘lead trainer’ to run the 4Ts course	A meeting room with options to present or broadcast content	Description of syllabus, objectives and timetable to send to delegates, including “basic ground rules”
Faculty size appropriate for number of delegates	Simulation training room if course face to face	E-learning modules available online
Suitable number of delegates	Suitable simulation equipment, for example, mannequins, hydrogel models and VR simulation	Procedural based courses should describe the important anatomy, port placement, and surgical tasks/steps and errors to avoid
	A list of equipment required for training exercises, for example, performance-enhancing instruction exercise (throwing a ball in a bucket behind you that you cannot see); see Sect. 5 for the list of exercises	Data analysis forms/outcome metrics/checklists
	Novel technologies to demonstrate e.g. VR headsets	Educational video examples of good and bad practice
	Functional Telepresence kit with live feed	Written vignette/scenarios for role play with stated aims of roleplay
		Team training examples with nontechnical skills training scenarios

The panel agreed that completion of E-learning related to the 4 T's course should be completed, with baseline evaluation of knowledge of the course subject matter and/or technical procedure aspects, before attending the 4 T's course. So that trainers and delegates have appropriate background knowledge and lead trainers can identify the participants gaps in knowledge of the program and address them before the 4 T's course commences. Additionally, baseline evaluation of the delegates should be benchmarked, once enough data from 4Ts courses are available to formulate a benchmark. This benchmark needs to be set and quantified so that it is specific to a course. Pre-course evaluation should include opportunity to provide details of what additional aspects the trainer delegates want to get from the 4 T's course. The panel also agreed it was advantageous that future appointed faculty trainers should have completed a 4Ts course as delegates.

#### *Section 5: Pedagogy and course content to be included*

The panel reached agreement on multiple areas of pedagogy and course content for a standardised 4Ts course. Below we summarise the areas of agreement related to subject matter, exercises and feedback.

Important subject matters to be covered in a 4Ts course:

1. Highlighting the importance of team contribution to training and describing the behaviours of “good” team members [28].
2. How to deal with the difficult trainee [29].
3. Guidance on how to avoid taking over in theatre as the trainer; explanation of the “six steps” of safe training in the OR [30]
4. Non-technical skills (cognitive and social competencies): situational awareness, decision-making, communication, teamwork and leadership [28].
5. Human factors: how teamwork, tasks, equipment, workspace, culture, and organization impact human behaviour and performance, with the goal of minimizing errors and enhancing clinical performance [31].
6. Definition of benchmarked metric-based progression [32]
7. The importance of reflection and the association with learning in conscious competence [33].
8. How to debrief [34]
9. How to give formative feedback [35]
10. Examples of digital assessment tools and when to use them [36].
11. How to use video performance assessment tools [37].
12. Task deconstruction with error training and recognition [38].
13. Description and explanation of “performance-enhancing feedback” [39]
14. Identification and optimisation of take-home messages

Exercises:

1. Practical demonstrations/role play/tasks/group participation
2. Task repetition to demonstrate deliberate practice [40].
3. Example of task deconstruction, defining a specific activity to be taught, reducing the activity to the smallest component parts [34].
4. Explanation of the “six steps”: (1) stop, (2) identify, (3) explain, (4) structured teaching, (5) elicit check of understanding, and (6) proceed if safe [30]
5. Metrics-based progression exercises [41]
6. Role play exercise that describes and explains the effect of cognitive load [42].
7. Formative feedback exercise (throwing a ball in the bucket behind you with formative feedback) [42].
8. Practical training roleplay in formative feedback: role play scenarios played out with delegate interaction and assessment of trainer's performance with open discussion and feedback.

#### *Section 6: Measuring outcomes*

The panel agreed that a 4 T's course learning should be metric based and benchmarked according to each course when sufficient data becomes available [16]. The effectiveness of the 4Ts course should undergo construct validation indicating that the assessments can differentiate between expert trainers and novices. Delegates should have the opportunity to repeat the assessments until they achieve the set benchmark, but it was highlighted that 4Ts courses should prioritise encouragement and support for delegate trainers above high stakes scores. It is also recommended that newly trained faculty have training and assessment in their knowledge and skills on using specific training technologies (e.g. VR/AR simulation) that are planned to be utilised in the training course. The panel also agreed that the robustness of Technical Skills assessment tools is important for the continuum of learning and big data analysis and future benchmark setting [10].

#### *Section 7: Integration of novel technologies to aid surgical training*

There was 100% agreement from the panel that in a modern robotic training curriculum, both knowledge and skills training can be aided with the integration of novel technologies such as VR simulation or automated data collection. There was 100% agreement that the trainer should be aware of the various educational technologies available to aid knowledge and skills acquisition and that the trainer should be trained in the various educational technologies available, for the robotic system they are training trainees. Table 4 summarises training technologies and their application in device training, basic skills training and procedural training. Technologies that have potential to collect automated data and AI analytics are highlighted in blue boxes (see Table 5).

**Table 5:**
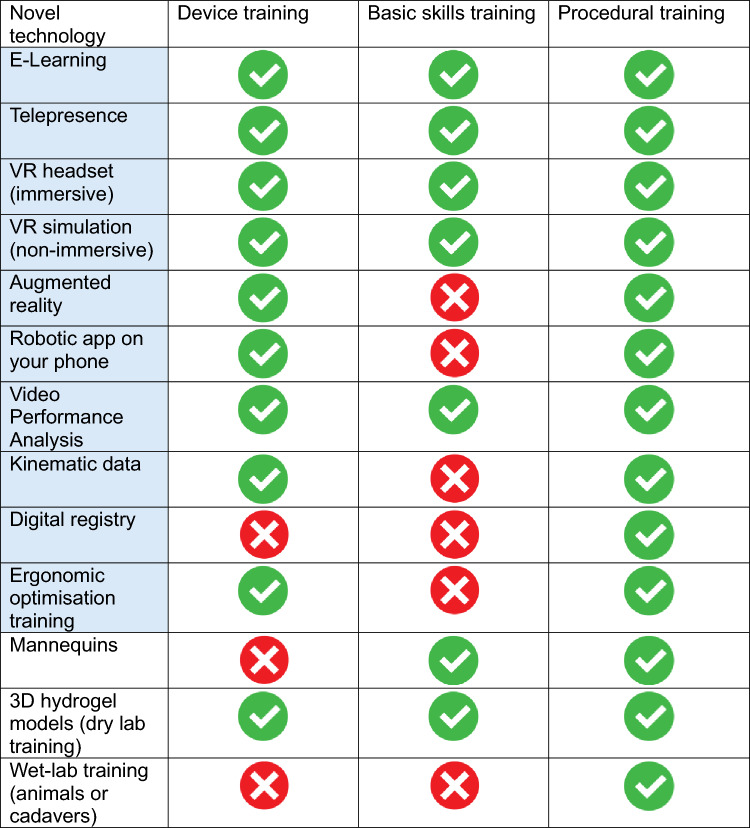
training technologies recommended in device training, basic skills training and procedural training

#### *Section 8: Regulation*

**Fig. 2:**
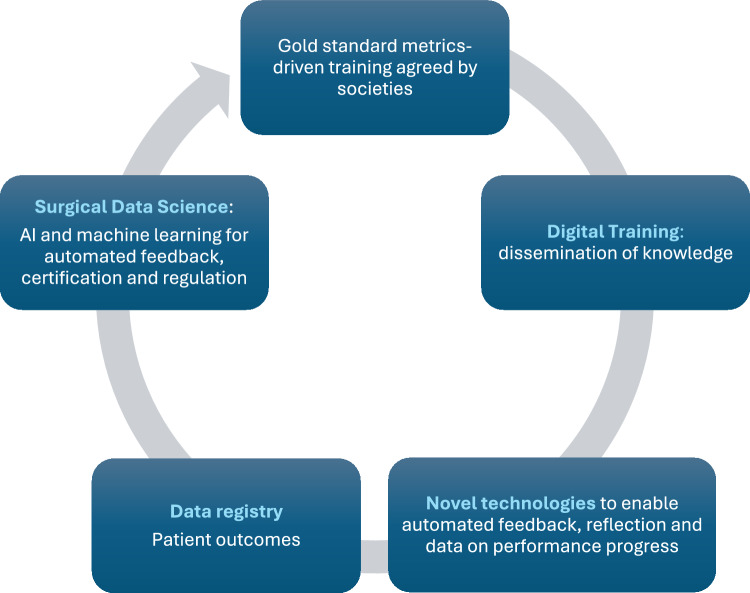
Enabling continual improvement by connecting data points from training to patient outcomes

The panel agreed that the 4Ts curriculum content, eligibility to be a lead trainer and suitable to be an approved trainer in procedural skills training should all be regulated by the relevant surgical societies. The findings are aligned with previous expert consensus that agreed that privileges should be granted based on video review of surgical performance and attainment of clearly defined objective proficiency benchmarks. Parameters for ongoing outcome monitoring were determined and recommendations for technical skills training, preceptorship, and performance assessment were defined [43] (Fig. 2).

## Discussion

Surgical robots are utilised in multiple soft tissue procedures in urologic, gynaecologic, general, cardiothoracic, and head and neck surgery as well as being used in orthopaedics [2]. Robotic surgery has great potential to enable surgeons to perform complex minimally invasive procedures with improved visualisation, increased precision, and enhanced dexterity. However, it is recognised that errors are more common early in the surgeons learning curve [4] and the combination of simultaneously learning about both technology and technique, on patients, has inherent patient safety risks if training is not optimised [44]. Trainees need device training and foundational skills training before embarking on procedural training [45]. Innovation is driving changes to curricula content development but also adding new complexities to training. This is the first validated train the trainer in robotic surgery that incorporates guidance on the integration of training technologies.

Digital transformation of robotic surgical training with integration of novel digital products can provide infrastructure and new opportunities for surgical data science (SDS) with data annotation, shared learning and data analytics [46]. SDS is the scientific discipline that seeks to improve surgical quality, safety, and the value of surgical intervention through the acquisition, organization, analysis, and modeling of data.

The opportunities from SDS and delivering scalable affordable training are huge. The best way to realise these opportunities is to educate and train trainers on how best to utilise technologies and then to educate trainees with the same insights, thereby empowering future generations of surgical trainees with access to surgical knowledge and skills on a global scale. A 4Ts course can enable this via a top-down approach with controlled dissemination of agreed reference performance metrics and benchmarks, that will aid data normalisation and quality assurance [10, 46].

Digital transformation of surgical training also offers new solutions to access training and to reward all stakeholders following the FAIR and CARE principles. FAIR (Findable, Accessible, Interoperability and Re-usable) and CARE (Collective benefit, Authority to Control, Responsible, Ethical). The FAIR and CARE principles are complementary sets of guidelines for data access, retrieval, usability and governance that aims to ensure data is handled in ways that are respectful to all the various stakeholders’ rights and interests [8].

Network development will aid access to experts’ insights, new knowledge and skills. Network group size dictates the amount of time an individual trainee receives, and consideration of the scalable approaches needed to accommodate the size of the audience who need to be trained and the availability of expertise in different settings. Unlimited numbers of trainees can connect via reusable online E-Learning, video libraries, social media apps and live streaming of content [47]. Smaller groups can benefit from virtual classrooms, which have been shown to be three times more efficient in surgical skills training delivered by trainers, than in face-to-face training. This was achieved with individual cameras per trainee and classroom screen setup where the trainer could monitor progress in up to 12 trainees at once [17]. Other efficiencies of scale can be achieved with courses that deliver platform agnostic training or basic skills training that is cross-specialty applicable [48]. Personalised one to one training with telementoring can deliver access to expertise whilst avoiding additional costs and novel telepresence services now include telepreceptorship and co-surgery [11, 49]. Telepresence services are also a rich supply for video data that can be collected and annotated, providing new opportunities for big data to combine with machine learning to deliver predictive analytics and automated performance feedback for trainees [50].

However, it is not sufficient to assume that big data and AI will solve all our training needs. There are also elements of training, such as non-technical skills (NTS), that will likely benefit from face-to-face training [4] and many successful elements of current analog training can be built on rather than discarded. There are other crucial factors directly related to trainers that can limit successful implementation of curricula, including trainers’ expertise as an educator, their knowledge and communication skills, and their familiarity with both curriculum content and awareness of the available novel technologies available to teach both technical and NTS. To improve the teaching performance of robotic-assisted surgery trainers, it is necessary to understand the important attributes of expert trainers, raise awareness of novel digital training technologies and optimal methods to deliver faculty development [4].

## Conclusions

Using the Delphi methodology, we achieved expert consensus involving academic, expert trainers and industry to develop and reach content validation for guidance on a 4Ts curriculum for robotic surgery training. This recommended content lays the foundation for developing platform agnostic metric-based progression curricula for trainers in robotic surgery, that raise awareness about SDS and optimises integration of digital training technologies. Future 4Ts curricula related to procedural training will require further validation.

## Data Availability

No datasets were generated or analysed during the current study.
