# Correction: Digital transformation of robotic surgery train the trainer ‘TTT’ courses: training the trainer in technique and technology (the 4Ts course)

**DOI:** 10.1007/s11701-025-02777-5

**Published:** 2025-09-29

**Authors:** Justin W. Collins, Nader Francis, Fares Haddad, Mark Coleman, Steven Stamenkovic, Asit Arora, Bas Wijnhoven, Altaf Awan, Tom Cecil, Nahid Gul, Jawad Ahmad, Subash Vasudevan, Ben Challacombe, Nuha A. Yassin

**Affiliations:** 1 Division of Surgery & Interventional Science, University College London, London, UK; 2 Division of Uro-Oncology, University College London Hospital, London, UK; 3 University College London, London, UK; 4 University Hospitals Birmingham NHS Foundation Trust, Birmingham, UK; 5 The University of Birmingham, Birmingham, UK; 6 The Royal College of Surgeons of England, London, UK; 7 The Griffin Institute, Northwick Park, Harrow, UK; 8 St Mark’s Hospital, London, UK; 9 Department of Surgery, University Hospitals Plymouth NHS Trust, Plymouth, United Kingdom; 10 Thoracic Surgery, Barts Health NHS Trust, London, United Kingdom; 11 ENT Surgery, Guy’s and St Thomas’ NHS Foundation Trust, London, United Kingdom; 12 Oncological and Gastrointestinal Surgery, Erasmus University Medical Center, Rotterdam, Netherlands; 13 Division of General Surgery, Derby Hospitals NHS Foundation Trust, Derby, United Kingdom; 14 Colorectal Surgery, Hampshire Hospitals NHS Foundation Trust, Basingstoke, United Kingdom; 15 Obstetrics and Gynaecology, Wirral University Teaching Hospital NHS Foundation Trust, Wirral, United Kingdom; 16 Hepatobilary Surgeon, University Hospitals Coventry & Warwickshire NHS Trust, Coventry, United Kingdom; 17 General Surgery, East Suffolk and North Essex NHS Foundation Trust, Colchester, United Kingdom; 18 Department of Urology, Guy’s and St Thomas’ NHS Foundation Trust, London, United Kingdom

**Correction: Journal of Robotic Surgery (2025) 19:510** 10.1007/s11701-025-02685-8

In the original version of this article, the affiliation details for the authors Tom Cecil and Subash Vasudevan were incorrectly given as

Tom Cecil

The Griffin Institute, Northwick Park, Harrow, UK

St Mark’s Hospital, London, UK

Colorectal Surgery, Hampshire Hospitals NHS Foundation Trust, Basingstoke, United Kingdom

Subash Vasudevan

The Griffin Institute, Northwick Park, Harrow, UK

St Mark’s Hospital, London, UK

General Surgery, East Suffolk and North Essex NHS Foundation Trust, Colchester, United Kingdom

But should have been

Tom Cecil

Colorectal Surgery, Hampshire Hospitals NHS Foundation Trust, Basingstoke, United Kingdom

Subash Vasudevan

General Surgery, East Suffolk and North Essex NHS Foundation Trust, Colchester, United Kingdom

